# Transforming Growth Factor β_1_ Oppositely Regulates the Hypertrophic and Contractile Response to β-Adrenergic Stimulation in the Heart

**DOI:** 10.1371/journal.pone.0026628

**Published:** 2011-11-17

**Authors:** Michael Huntgeburth, Klaus Tiemann, Robert Shahverdyan, Klaus-Dieter Schlüter, Rolf Schreckenberg, Marie-Luise Gross, Sonja Mödersheim, Evren Caglayan, Jochen Müller-Ehmsen, Alexander Ghanem, Marius Vantler, Wolfram H. Zimmermann, Michael Böhm, Stephan Rosenkranz

**Affiliations:** 1 Klinik III für Innere Medizin, Herzzentrum der Universität zu Köln, Cologne, Germany; 2 Abteilung für Kardiologie und Angiologie, Universitätsklinikum Münster, Münster, Germany; 3 Institut für Physiologie, Justus-Liebig-Universität Gießen, Gießen, Germany; 4 Institut für Pathologie, Ruprecht-Karls-Universität Heidelberg, Heidelberg, Germany; 5 Center for Molecular Medicine Cologne (CMMC), Universität zu Köln, Cologne, Germany; 6 Medizinische Klinik und Poliklinik II, Universitätsklinikum Bonn, Bonn, Germany; 7 Abteilung für Pharmakologie, Georg August Universität Göttingen, Göttingen, Germany; 8 Klinik für Innere Medizin III, Universität des Saarlandes, Homburg/Saar, Germany; University of Chicago, United States of America

## Abstract

**Background:**

Neuroendocrine activation and local mediators such as transforming growth factor-β_1_ (TGF-β_1_) contribute to the pathobiology of cardiac hypertrophy and failure, but the underlying mechanisms are incompletely understood. We aimed to characterize the functional network involving TGF-β_1_, the renin-angiotensin system, and the β-adrenergic system in the heart.

**Methods:**

Transgenic mice overexpressing TGF-β_1_ (TGF-β_1_-Tg) were treated with a β-blocker, an AT_1_-receptor antagonist, or a TGF-β-antagonist (sTGFβR-Fc), were morphologically characterized. Contractile function was assessed by dobutamine stress echocardiography *in vivo* and isolated myocytes *in vitro*. Functional alterations were related to regulators of cardiac energy metabolism.

**Results:**

Compared to wild-type controls, TGF-β_1_-Tg mice displayed an increased heart-to-body-weight ratio involving both fibrosis and myocyte hypertrophy. TGF-β_1_ overexpression increased the hypertrophic responsiveness to β-adrenergic stimulation. In contrast, the inotropic response to β-adrenergic stimulation was diminished in TGF-β_1_-Tg mice, albeit unchanged basal contractility. Treatment with sTGF-βR-Fc completely prevented the cardiac phenotype in transgenic mice. Chronic β-blocker treatment also prevented hypertrophy and ANF induction by isoprenaline, and restored the inotropic response to β-adrenergic stimulation without affecting TGF-β_1_ levels, whereas AT_1_-receptor blockade had no effect. The impaired contractile reserve in TGF-β_1_-Tg mice was accompanied by an upregulation of mitochondrial uncoupling proteins (UCPs) which was reversed by β-adrenoceptor blockade. UCP-inhibition restored the contractile response to β-adrenoceptor stimulation *in vitro* and *in vivo*. Finally, cardiac TGF-β_1_ and UCP expression were elevated in heart failure in humans, and UCP – but not TGF-β_1_ – was downregulated by β-blocker treatment.

**Conclusions:**

Our data support the concept that TGF-β_1_ acts downstream of angiotensin II in cardiomyocytes, and furthermore, highlight the critical role of the β-adrenergic system in TGF-β_1_-induced cardiac phenotype. Our data indicate for the first time, that TGF-β_1_ directly influences mitochondrial energy metabolism by regulating UCP3 expression. β-blockers may act beneficially by normalizing regulatory mechanisms of cellular hypertrophy and energy metabolism.

## Introduction

Transforming growth factor-β_1_ (TGF-β_1_) is a 25-kDa homodimeric protein that is involved in numerous cellular processes [1], [2]. In the heart, TGF-β_1_ is expressed at high levels during embryonic development and pathology [3]–[6]. Both, TGF-β_1_ ligand and its two serine-threonine kinase receptors, termed TGF-β receptor type I and II (TβRI and TβRII), are present in cardiac tissue, and all are expressed in cardiac myocytes and non-myocytes [4]–[6]. TGF-β_1_ has been implicated in a number of cardiac diseases such as pressure overload hypertrophy, post myocardial infarction ventricular remodeling, idiopathic hypertrophic cardiomyopathy, and dilative cardiomyopathy [5], [6]. In particular, TGF-β_1_ is highly expressed in hypertrophic myocardium during the transition from stable hypertrophy to heart failure [7], indicating that it may play a role in the functional deterioration of the hypertrophied heart.

Ventricular remodeling is a dynamic process of alterations in size, shape and function of the left ventricle that involves adaptive and/or pathologic changes of cardiac myocytes and interstitial tissue. It is well established that activation of neuroendocrine mechanisms such as the renin-angiotensin-aldosterone system (RAAS) and the sympathetic nervous system as well as the induction of local mediators contribute to the structural and functional alterations in the hypertrophied heart [5]–[12]. TGF-β_1_ may be a crucial mediator of cardiac remodeling through direct and indirect actions in cardiomyocyte hypertrophy, fibroblast proliferation, and extracellular matrix metabolism [5], [6]. While increased TGF-β_1_ levels were associated with cardiac hypertrophy and fibrosis [13], [14], the loss of one TGF-β_1_ allele in heterozygous TGF-β_1_ deficient mice resulted in decreased fibrosis of the aging heart [15]. An extensive body of evidence suggests a direct functional association between TGF-β_1_, the RAAS and the β-adrenergic system. Several studies have demonstrated that angiotensin II induces TGF-β_1_ mRNA and protein expression in cardiac myocytes and fibroblasts via the angiotensin type 1 (AT_1_) receptor *in vitro* [4]–[6], and appears to be required for angiotensin-induced cardiac hypertrophy *in vivo* [16]. In addition, TGF-β_1_ was shown to modulate the number and function of β-adrenergic receptors in various cell types [5], [17], and to alter β-adrenergic signaling in the heart *in vivo* [13], [18]. However, the precise interplay and the functional consequences of the network involving TGF-β_1_, the RAAS, and the β-adrenergic system have not been thoroughly characterized.

To better understand the mechanisms by which TGF-β_1_ induces cardiac fibrosis and hypertrophy, and furthermore may contribute to myocardial dysfunction, we took advantage of a transgenic mouse model that overexpresses a mature form of TGF-β_1_. Based on the well established connection between TGF-β_1_, the RAAS and the β-adrenergic system, TGF-β_1_ transgenic mice were chronically treated with a β-adrenoceptor blocker, an angiotensin AT_1_ receptor antagonist, or an antibody against the TGF-β receptor. The results identify TGF-β_1_ as an important regulator of cardiomyocyte growth and function. Furthermore, our data suggest that the β-adrenergic system is critically involved in TGF-β_1_-induced cardiac phenotype, as TGF-β_1_ promotes the hypertrophic responsiveness to β-adrenergic stimulation, whereas it impairs the contractile response to β-adrenergic stimuli by affecting the energy metabolism in the heart.

## Materials and Methods

### Animals and treatment

Alb/TGF-β_1_(cys^223,225^ser) transgenic mice were generated and maintained as described [13], [19]. The TGF-β_1_ cDNA encodes cysteine-to-serine substitutions at amino acid residues 223 and 225, resulting in preferential secretion of mature TGF-β_1_ [20]. Mice were treated from week three (immediately after weaning) to week 8 with either metoprolol (350 mg/kgBW/d) or telmisartan (10 mg/kgBW/d), each supplied with the drinking water, or by intraperitoneal application of soluble TGF-β receptor-Fc (sR-Fc; 1 mg/kgBW every other day). The latter compound was previously shown to act as a potent TGF-β antagonist [21]. All investigations were carried out at the age of 8 weeks, and all animal studies were performed according to NIH and Institutional animal care and use guidelines, and were approved by the local animal care authorities.

### Human heart tissue

Left ventricular tissue was obtained from explanted hearts of patients with dilative cardiomyopathy (DCM) undergoing heart transplantation. Groups consisted of patients without beta-blocker treatment (DCM) and with beta-blocker (metoprolol) treatment. In DCM hearts, left ventricular ejection fraction (LVEF) was <40%. Non-failing controls represent healthy donor hearts that could not be implanted for whatever reason. The use of myocardial tissue samples was approved by the local ethics committee, and written informed consent was obtained from all patients.

### Tissue homogenization, isolation of mitochondria and Western blot analyses

Left ventricular tissue was homogenized by incubation in extraction buffer (10 mM cacodylic acid, 150 mM NaCl, 1 µM ZnCl_2_, 20 mM CaCl_2_, 1.5 mM NaN_3_, 0.01% Triton-X100, pH 5.0) for 12 h at 4°C and subsequent centrifugation for 10 min at 1,200× g. For isolation of mitochondria, fresh tissue was repeatedly minced in ice-cold STE, chopped in ice-cold STE buffer (250 mM sucrose, 5 mM Tris, 2 mM EGTA, pH 7.4 at 4°C) and rinsed. The chopped tissue was subjected to proteinase digest and disrupted in a Dounce homogenizer with a tight plunger. The homogenate was centrifuged at 700× g for 10 minutes at 4°C, the supernatant was filtered through cheese cloth and centrifuged at 8,000× g for 10 minutes. The pellet was resuspended in 40 µl of RIPA-buffer, subjected to sonication and the mitochondrial protein content was assayed using the bicinchonic acid method (BioRad). Similar amounts of protein were resolved on a 10% SDS-polyacrylamide electrophoresis gel, and the proteins were transferred to Immobilon and subjected to Western blot analysis using antibodies that recognize TGF-β_1_ (R&D), RasGAP, UCP-3 (Affinity BioReagents), or cytochrome *c* oxidase complex IV (COX-I; Invitrogen).

### Morphometric analysis of myocardial tissue

Fixation of myocardial tissue was performed by retrograde perfusion fixation as previously described [22]. Animals were perfused with either ice-cold NaCl 0.9% (immunohistochemistry) or 3% glutaraldehyde (morphometric and stereological analysis). Routine tissue stains were obtained from transversally cut hearts that were either fixed in 4% buffered formalin or snap-frozen in liquid nitrogen. After paraffin sections were embedded, they were stained with hematoxylin and eosin. Tissue sampling and section staining were performed according to the orientator method [23]. Fractional areas of cardiac myocytes, cardiac fibroblasts, and interstitium were measured on 12 differentially orientated semithin sections per animal using the point-counting method [22]. Myocyte diameters were measured on longitudinal sections with a semiautomatic image analyzing system and corrected for sarcomere length.

### RNA isolation and quantitative real-time PCR

For UCP mRNA expression analyses, total RNA was isolated using the RNeasy Fibrous Tissue Mini Kit (Qiagen). cDNA was synthesized using the SuperScript III first-strand synthesis system with random hexamers (Invitrogen). For expression analyses of atrial natriuretic factor (ANF) and ornithine decarboxylase (ODC), mouse hearts were isolated and perfused in the Langendorff-mode. A subset was stimulated with isoprenaline (1 µM), and perfusion was prolonged for 2 hours. Release of lactate dehydrogenase was monitored to control for sarcolemmal integrity. Subsequently, ventricular tissue was dissected and RNA was extracted using RNA-Clean (AGS, Heidelberg, Germany). Total RNA from cultured cardiomyocytes was isolated using the TRIzol method (Invitrogen). Reverse transcription was performed using Sensiscript reverse transcriptase (Qiagen) and oligo-dt primers for ANF, ODC, and β-actin as described [13], [18]. Quantitative real-time PCR was performed using TaqMan gene expression assays (Applied Biosystems) or SYBR Green Master Mix as indicated. Primers used are listed in table 1. Relative abundance of the gene of interest was calculated after normalization to 18S ribosomal RNA or β-actin as indicated.

**Table 1:** Oligonucleotide sequences for primer and probe sets for rat cardiomyocytes.

UCP2 forward primer	5′-TCATCAAAGATACTCTCCTGAAAGC-3′
UCP2 reverse primer	5′-TGACGGTGGTGCAGCAGA-3′
UCP2 probe	5′-FAM-TGACAGACGACCTCCCTTGCCACT-TAMRA-3′
UCP3 forward primer	5′-GTGACCTATGACATCATCAAGGA-3′
UCP3 reverse primer	5′-GCTCCAAAGGCAGAGACAAAG-3′
UCP3 probe	5′-FAM-CTGGACTCTTCACCTGTTCACTGACAACTTCC-TAMRA-3′

### Dobutamine stress echocardiography

Images were obtained by using a HDI-5000 ultrasound device (Philips Medical Systems, Bothell, WA, USA) equipped with a linear array transducer (15 MHz) as described [24]. At least 20 cardiac cycles were obtained for each view, and each imaging plane was acquired three times to assess reproducibility. Parasternal short-axis views were divided into six segments, and long axis views were divided into seven segments [25]. The endocardial borders were manually traced on the innermost endocardial edge while the epicardial borders were defined by tracing along the first bright pixel adjacent to myocardial tissue [26]. Left ventricular mass (LVM) and LVEF were assessed as previously described [24]. The resistive index (RI) was calculated as 1 – enddiastolic velocity/systolic velocity. Dobutamine was administered intravenously at 10, 20, and 40 µg/kgBW/min after microscopical cannulation of the tail vein. This procedure corresponds to the recommended protocol of the *American Society of Echocardiography* that is used in humans [27]. 2D- and M-mode registrations were recorded at each level of dobutamine.

### Contractility of isolated cardiac myocytes

Cardiac myocytes were isolated by standard procedures as described [28]. Briefly, mouse hearts were exposed to collagenase digest in the Langendorff-mode, minced, and further digested by incubation with collagenase buffer. The suspension was filtered, and cardiomyocytes were separated from non-myocytes by centrifugation. Finally, physiological calcium concentrations were readjusted by step-wise increases to 1000 nmol/l, and plated on laminin-coated culture dishes. Cell contraction was investigated using a cell-edge detection system as previously described [29]. Briefly, cells were stimulated with biphasic electrical stimuli composed of two equal but opposite rectangular 50-V stimuli of 0.5 ms duration. Each cell was stimulated at 1, 0.5, and 2 Hz for 1 min. Every 15 s the next five contractions were averaged. The mean of these four measurements at a given frequency was used to define the contractility of a given cell. Cell lengths were measured at a rate of 500 Hz via a line camera.

### Statistical analyses

All data are expressed as means ± SEM. Statistical significance was estimated by ANOVA, followed by post-hoc analysis (Student-Neuman-Keuls test), or by using the Student's t-test for paired or unpaired observations, as appropriate. A *p* value of less than 0.05 was considered statistically significant.

## Results

### Cardiac hypertrophy in TGF-β_1_ transgenic mice is prevented by chronic β- adrenoceptor blockade

**Figure 1:**
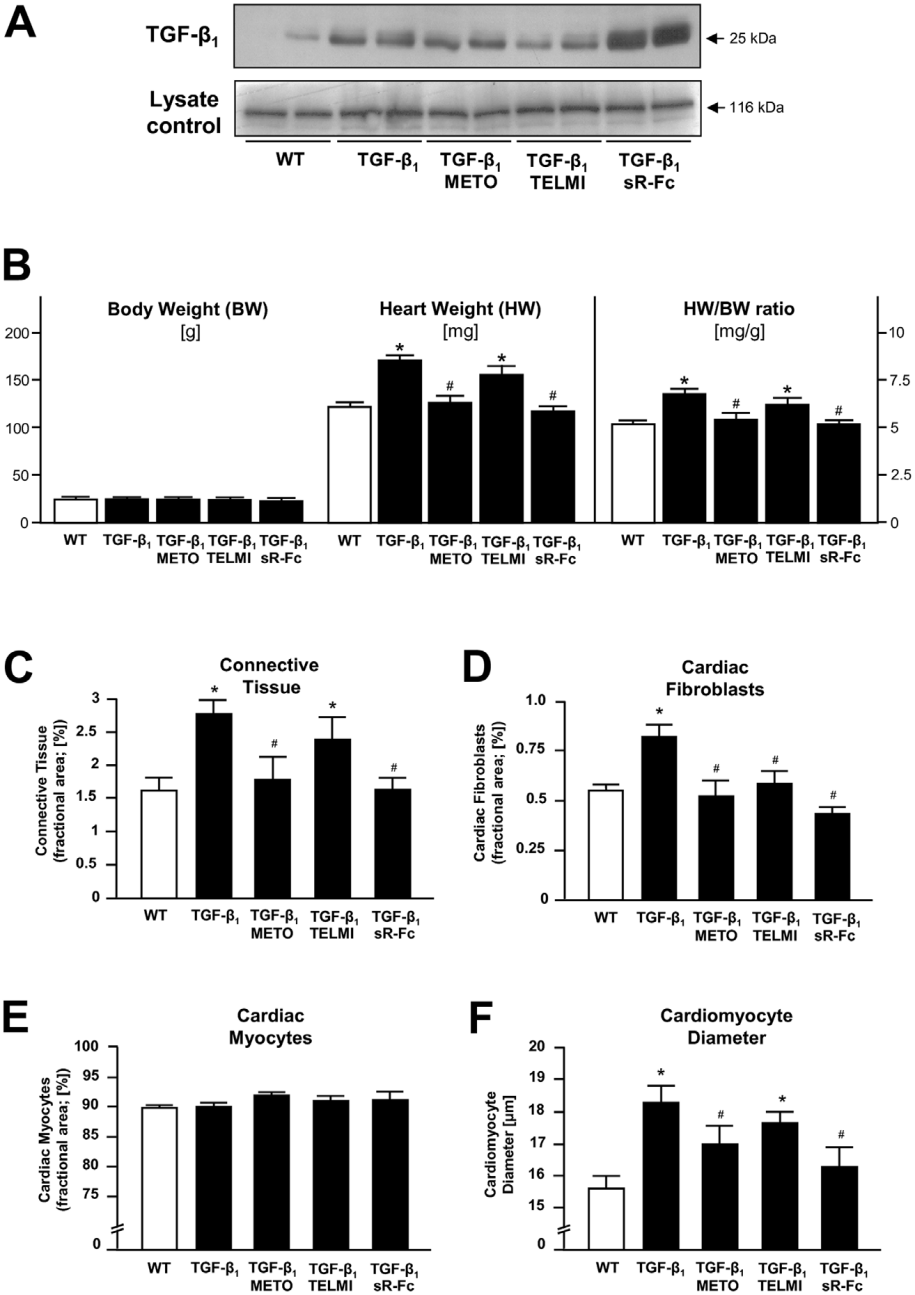
Characterization of myocardial tissue in wild type (WT) and TGF-β_1_ transgenic mice (TGF-β_1_) that have been treated with either metoprolol (METO), telmisartan (TELMI), or soluble TGF-βR-Fc (sR-Fc). (**A**) Myocardial TGF-β_1_ protein expression as determined by Western blotting in heart homogenates from the various groups as indicated. RasGAP served as lysate control. (**B**) Body weight, heart weight, and heart/body weight ratio (n = 30–57 animals in each group). (**C–F**) Morphometric analysis of myocardial tissue (n = 5–9 animals in each group). Shown are the fractional areas of connective tissue (**C**), cardiac fibroblasts (**D**), cardiac myocytes (**E**), and cardiomyocyte diameter (**F**). *p<0.05 vs. WT, ^#^ *p*<0.05 vs. untreated TGF-β_1_ mice.

Compared to wild-type mice, transgenic mice overexpressing a mature form (cys^223,225^ser) of TGF-β_1_ (Fig. 1A) displayed cardiac hypertrophy, as indicated by an increase in heart weight (170.5±3.4 vs. 122.3±3.4 mg; *p*<0.05) with no change in body weight, resulting in a significant increase of the heart-to-body-weight ratio (6.8±0.1 vs. 5.1±0.1 mg/g; *p*<0.05; Fig. 1B). The data on cardiac hypertrophy in TGF-β_1_ transgenic mice have been published previously [13] and are included here for completeness. Cardiac hypertrophy was not due to hypertension as the invasively measured mean arterial blood pressure was similar in wild type and transgenic mice (99.7±4.2 vs. 98.9±4.3 mmHg, n = 7 in each group, n.s.). In order to investigate whether the TGF-β_1_-induced cardiac phenotype involved neuroendocrine activation, TGF-β_1_ transgenic mice were chronically treated with the β-adrenoceptor blocker metoprolol, the angiotensin AT_1_ receptor antagonist telmisartan, or a TGF-β antagonist (sR-Fc). While neither treatment affected the elevated TGF-β_1_ protein levels in transgenic mice (Fig. 1A), chronic application of either metoprolol or sR-Fc prevented the increase of the heart-to-body-weight ratio (5.3±0.3 and 5.1±0.2 mg/g, respectively; both *p*<0.05 vs. TGF-β_1_) whereas telmisartan did not prevent cardiac hypertrophy in transgenic mice (Fig. 1B).

Morphometric analysis of the hearts revealed that the increase in cardiac size of TGF-β_1_ transgenic mice was due to both fibrosis and myocyte hypertrophy (Fig. 1C–F). This is demonstrated by a significant increase in the fractional areas of connective tissue and cardiac fibroblasts (Fig. 1C and D), with no change in myocyte fractional area (Fig. 1E), and a significant increase of cardiomyocyte diameter (Fig. 1F). These morphological changes in TGF-β_1_ transgenic mice were prevented by β-adrenoceptor blockade with metoprolol and application of sR-Fc, but not by AT_1_ receptor blockade with telmisartan.

### TGF-β_1_ enhances the hypertrophic responsiveness to βAR agonists

**Figure 2:**
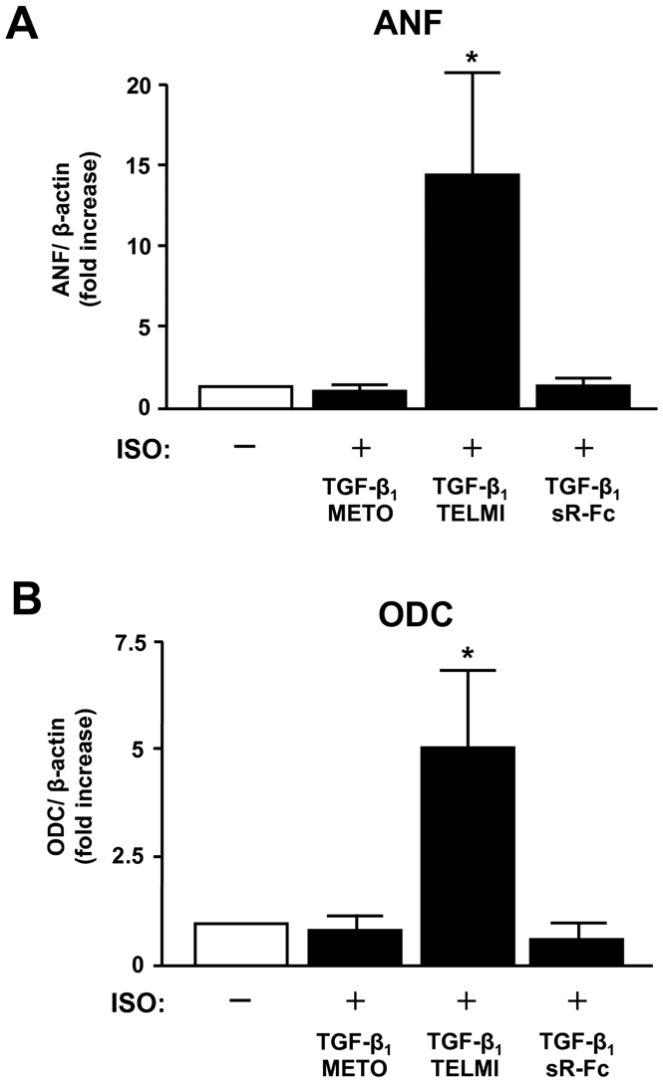
Induction of (A) atrial natriuretic factor (ANF) and (B) ornithine decarboxylase (ODC) mRNA by isoprenaline in hearts from TGF-β_1_ transgenic mice (TGF-β_1_) that have been treated with either metoprolol (METO), telmisartan (TELMI), or soluble TGF-βR-Fc (sR-Fc). Data are expressed as fold-increase relative to saline-perfused hearts. *p<0.05 vs. WT mice.

In adult heart muscle cells, cardiomyocyte hypertrophy upon adrenergic stimulation *in vitro* is exclusively mediated via the α-adrenergic receptor [30]. However, our previous studies have shown that cardiac hypertrophy in TGF-β_1_ transgenic mice is accompanied by an increased cardiac expression of hypertrophy-associated genes such as ANF which is further inducible by β-adrenergic stimulation in hearts from TGF-β_1_ but not from wild-type mice [17], [18]. This induction specifically depended on upregulation of ornithine decarboxylase (ODC), the rate limiting enzyme of the polyamine metabolism [18]. In line with the antihypertrophic effect of metoprolol as shown above, chronic β-adrenoceptor blockade as well as TGF-β antagonism prevented the induction of both ANF and ODC in isoprenaline-perfused hearts from TGF-β_1_ transgenic mice (Fig. 2A and B). In contrast, blockade of the angiotensin AT_1_ receptor did not prevent the induction of ANF and ODC in this model.

### TGF-β_1_ abrogates the contractile response to β-adrenergic stimulation

**Figure 3:**
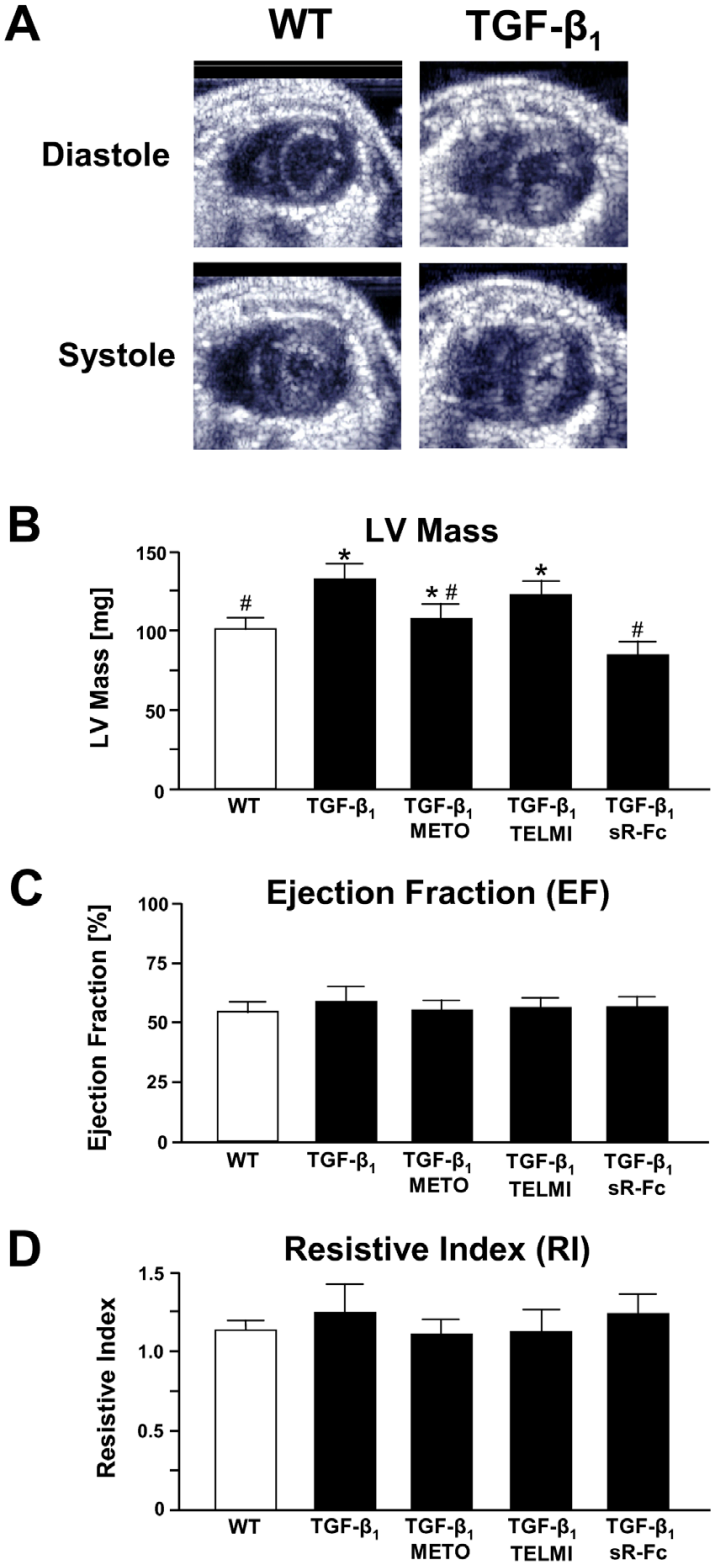
Echocardiographic evaluation of wild type (WT) and TGF-β_1_ transgenic mice (TGF-β_1_) that have been treated with either metoprolol (METO), telmisartan (TELMI), or soluble TGF-βR-Fc (sR-Fc). (**A**) Representative short axis views during diastole and systole in WT and TGF-β_1_ mice. (**B**) Left ventricular mass. (**C**) Left ventricular ejection fraction at rest. (**D**) Resistive Index. Data in B–D represent means ± SEM from 5–7 animals in each group. *p<0.05 vs. WT; ^#^ *p*<0.05 vs. untreated TGF-β_1_ mice.

Echocardiography confirmed cardiac hypertrophy in TGF-β_1_ mice *in vivo*, as LVM was increased in TGF-β_1_ mice compared to wild type animals (131.3±11.3 vs. 103.43±5.92 mg; *p*<0.05). Similar to gravimetrical analyses of the hearts, chronic treatment with metoprolol and sR-Fc prevented cardiac hypertrophy (LVM 109.5±11.0 and 82.8±6.3 mg, respectively; both *p*<0.05 vs. TGF-β_1_), whereas telmisartan did not (Fig. 3A and B). Under basal conditions, there was no difference in LVEF between wild-type and TGF-β_1_ mice (56.2±3.6 vs. 61.8±6.7%; n.s.), and none of the treatments had a significant effect on systolic function (Fig. 3C). Likewise, the resistive index (RI) as a measure of LV afterload was similar in all groups (Fig. 3D).

**Figure 4:**
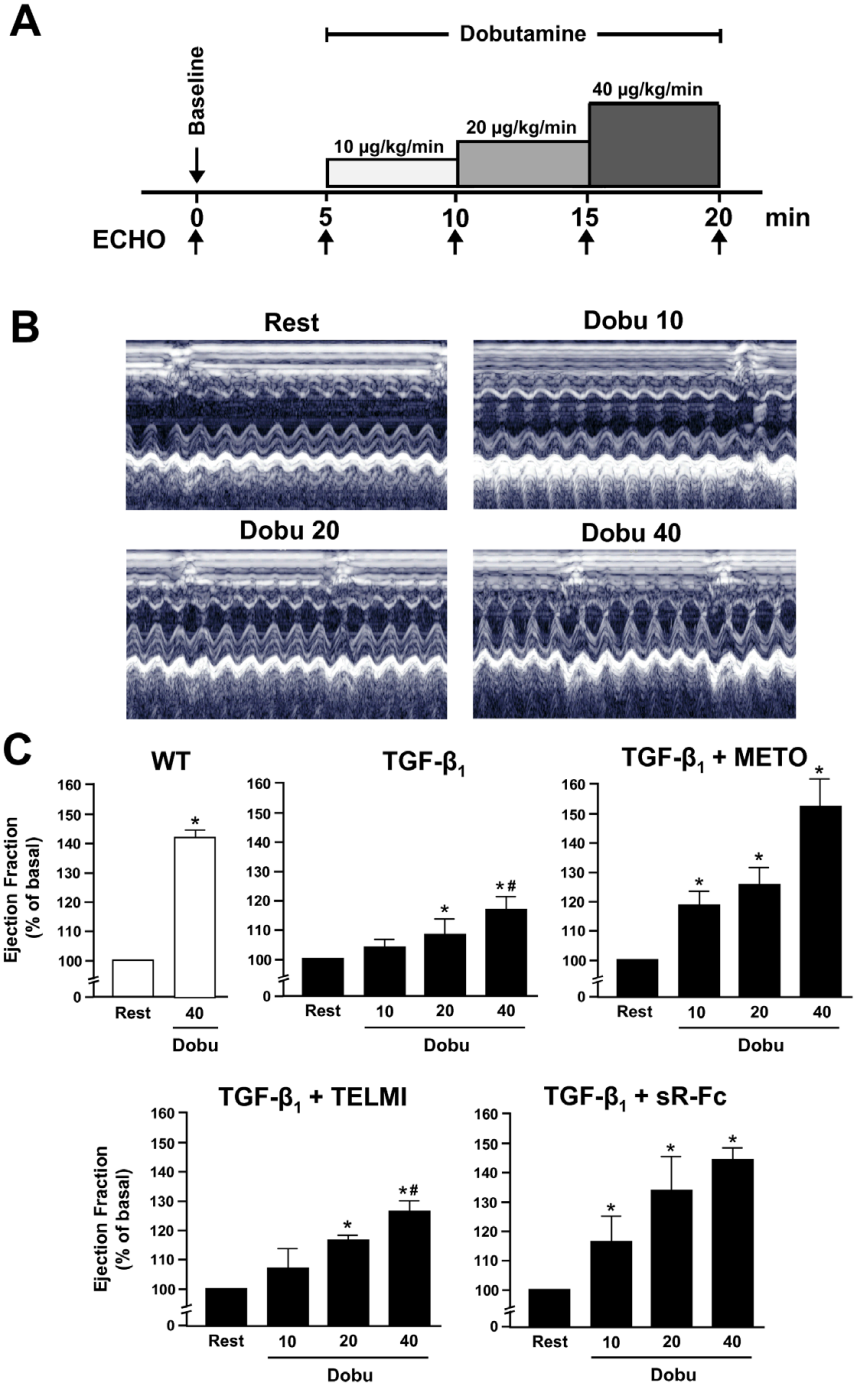
Dobutamine stress echocardiography (DSE). (**A**) DSE protocol as applied in mice. (**B**) Representative m-mode registrations in a WT mouse at rest, and at various concentrations of dobutamine (Dobu). (**C**) Contractile reserve in response to cumulative concentrations of dobutamine in wild type (WT) and TGF-β_1_ transgenic mice (TGF-β_1_) that have been treated with either metoprolol (METO), telmisartan (TELMI), or soluble TGF-βR-Fc (sR-Fc), n = 5–6 in each group. *p<0.05 vs. rest; ^#^ *p*<0.05 vs. WT.

In order to investigate the influence of TGF-β_1_ overexpression and the various pharmacological interventions on the contractile response to β-adrenergic stimuli, we performed dobutamine stress echocardiography (DSE) *in vivo* (Fig. 4A and B), and furthermore measured cell shortening of isolated cardiomyocytes *in vitro*. DSE revealed that the inotropic responsiveness to β-adrenergic stimulation was significantly diminished in TGF-β_1_ mice. At peak stress (dobutamine 40 µg/kg/min), the relative increase of LVEF was 16±5% in TGF-β_1_ mice vs. 44±5% in wild-type control mice (*p*<0.01). Both metoprolol and sR-Fc completely restored the inotropic responsiveness to dobutamine (relative increase in LVEF 52±10 and 43±5%, respectively; both *p*<0.05 vs. TGF-β_1_), whereas telmisartan had no significant effect (Fig. 4C).

**Figure 5:**
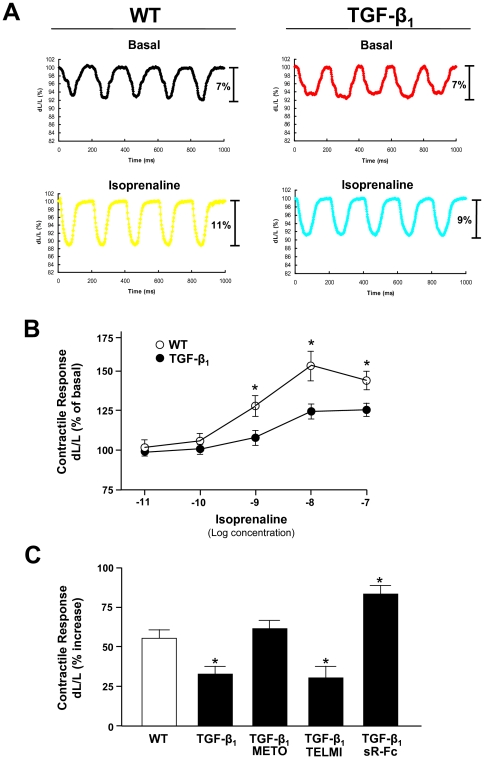
Contractility of isolated cardiac myocytes from wild type (WT) and TGF-β_1_ transgenic (TGF-β_1_) mice. (**A**) Representative original registrations of cell shortening (dL/L) under basal conditions and upon isoprenaline stimulation (10 µM). (**B**) Contractile response of isolated cardiomyocytes from WT and TGF-β_1_ mice to increasing concentrations of isoprenaline (n = 100 in each group). (**C**) Contractile response of isolated cardiomyocytes to 10 µM isoprenaline in the various treatment groups (n = 97–135 in each group). *p<0.05 vs. WT.

To further extend these findings to isolated cells, cardiac myocytes were isolated from wild-type and TGF-β_1_ transgenic mice that had been treated with the various compounds. There was no difference in basal contractility between cells from wild-type and TGF-β_1_ mice. Consistent with the data obtained by DSE, the contractile response of isolated cardiac myocytes to β-adrenergic stimulation was diminished in TGF-β_1_ transgenic mice (Fig. 5A and B), and was restored by chronic treatment with either metoprolol or sR-Fc, but not by telmisartan (Fig. 5C).

### Mitochondrial uncoupling proteins are involved in the reduction of contractile reserve in TGF-β_1_ transgenic mice

**Figure 6:**
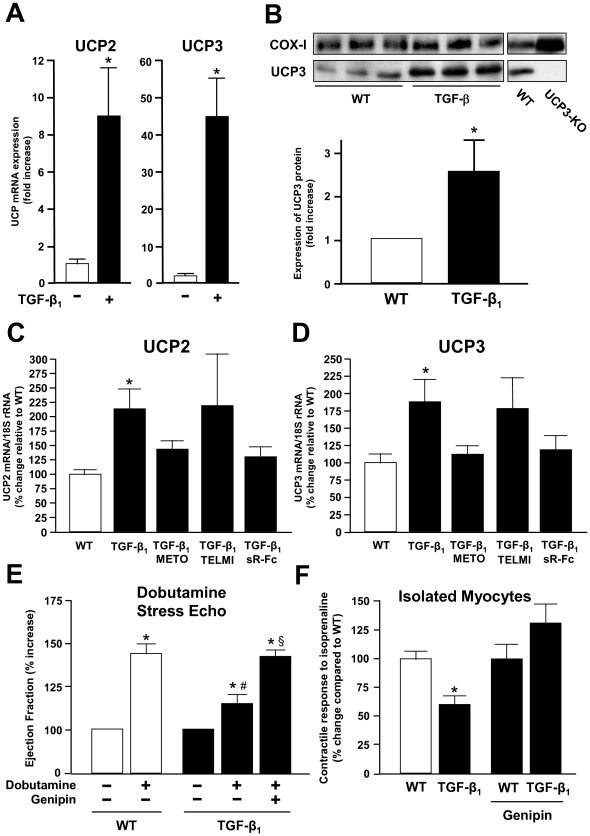
A role for uncoupling proteins (UCPs) for the diminished contractile reserve in TGF-β_1_ transgenic mice. (**A**) Stimulation of rat cardiac myocytes with TGF-β_1_ (10 ng/ml) leads to upregulation of UCP2 and UCP3 mRNA (n = 4 in each group). (**B**) Western blot analysis of UCP3 expression in mitochondria isolated from myocardial tissue of WT and TGF-β_1_ mice. COX-I served as a loading control, and UCP3 knockout mice served as a negative control. The bar graph represents means ± SEM from 7 animals in each group. (**C** and **D**) Expression of UCP2 and UCP3 mRNA in the various treatment groups. (**E**) Functional role of UCPs in the heart. Inhibition of UCPs by genipin (100 mg/kgBW) restored the contractile response to dobutamine (40 µg/kg/min) in TGF-β_1_ mice. (**F**) Genipin (5 µM) restored the contractile response to isoprenaline (10 µM) in isolated cardiac myocytes (n = 26–30 in each group).

Since our previous studies have demonstrated that hearts from TGF-β_1_ mice displayed an increased hypertrophic responsiveness to β-adrenergic stimulation and an increased contractility of atrial tissue when compared to wild-type mice [13], the fact that the contractile response in left ventricular tissue was diminished appeared surprising and warranted further investigation. To this end, we focused on mitochondrial uncoupling proteins (UCPs), which are involved in the regulation of energy metabolism in muscle cells by dissipating the proton gradient in the inner mitochondrial membrane, thereby causing a “proton leak” that results in reduced generation of ATP [31]. Our *in vitro* studies demonstrated that stimulation of rat cardiac myocytes with TGF-β_1_ led to a dramatic upregulation of UCP2 and UCP3 mRNA (Fig. 6A). Consistently, we found that the protein levels of UCP3 were significantly elevated in cardiac mitochondria that were isolated from TGF-β_1_ transgenic mice as compared to wild-type animals (Fig. 6B). In contrast, the protein levels of mitochondrial adenosine nucleotide transporter (ANT) and sarco-/endoplasmatic reticulum calcium-ATPase (SERCA) were not altered in the hearts of TGF-β_1_ transgenic mice (not shown). When the cardiac UCP levels in the various treatment groups were compared, chronic β-adrenoceptor blockade with metoprolol and TGF-β antagonism by sR-Fc, but not AT_1_ receptor blockade with telmisartan, significantly reduced the elevated mRNA levels of UCP2 and UCP3 in TGF-β_1_ mice (Fig. 6C and D), and this correlated with the ability of whole hearts and cardiac myocytes to adequately respond to β-adrenergic stimuli (see Figures 4 and 5). Downregulation of myocardial UCPs by β-adrenoceptor blockade occurred on the cellular level, as the induction of UCP2 and UCP3 by TGF-β_1_ in isolated cardiomyocytes was abolished by pre-treatment of the cells with metoprolol (not shown).

In order to assess whether UCPs are indeed involved with the reduced inotropic reserve in TGF-β_1_ mice, we finally applied a pharmacological compound, genipin, which was previously shown to act as a potent UCP inhibitor [32]. When TGF-β_1_ transgenic mice were injected with genipin (100 mg/kgBW i.p.), the contractile response to dobutamine during DSE was restored and comparable to the response of wild-type mice (relative increase of LVEF 42±4 vs. 44±5%; Fig. 6E). Furthermore, while the contractile response of isolated cardiac myocytes to isoproterenol was diminished in cells from untreated TGF-β_1_ mice, pre-treatment with genipin (5 µM) reversed this effect, so that cells from TGF-β_1_ mice responded better to isoproterenol than cells from wild-type mice (Fig. 6F). These data indicate that the expression and activity of UCPs are critically involved in the determination of the contractile response to β-adrenoceptor stimulation, and suggest that chronic β-blocker treatment improves the inotropic reserve in TGF-β_1_ mice by down-regulating the increased UCP levels.

### TGF-β_1_ and UCP3 expression in human heart failure

**Figure 7:**
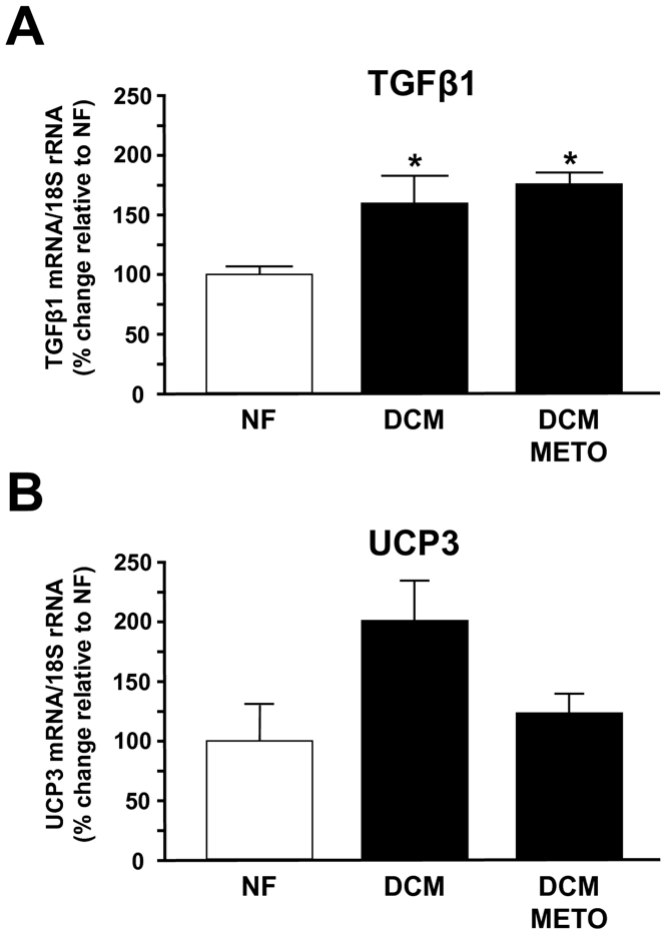
TGF-β_1_ (A) and UCP3 (B) expression in human heart. Myocardial samples were obtained from non-failing myocardium (NF; n = 3), and from DCM hearts of patients who had not received β-blocker treatment (DCM; n = 5) or patients who were treated with metoprolol (DCM-METO; n = 3). *p<0.05 vs. DCM.

To investigate whether this mechanism may also be relevant in humans, we measured TGF-β_1_ and UCP3 expression in non-failing hearts and in myocardium from patients with DCM who had either been treated or not been treated with metoprolol. In a limited number of human samples that was available to us, we show that TGF-β_1_ expression was increased in DCM hearts regardless of β-blocker treatment (Fig. 7A). Interestingly, there was a clear trend towards an increased expression of UCP3 in DCM hearts of patients who had not received metoprolol as compared to non-failing myocardium. In contrast to the expression levels of TGF-β_1_, there was lower UCP3 expression in DCM hearts from metoprolol-treated patients as compared to those who had not received metoprolol (Fig. 7B). Hence, increased expression of myocardial UCPs and its downregulation by β-adrenoceptor blockade is found at least in some patients with heart failure.

## Discussion

In this manuscript, we demonstrate that cardiac hypertrophy in mice overexpressing a mature form of TGF-β_1_ is accompanied by the induction of hypertrophic responsiveness to β-adrenergic stimulation, whereas the contractile β-adrenergic response in LV was diminished. Further analyses revealed that TGF-β_1_ impairs the inotropic reserve via regulation of mitochondrial UCPs which determine the efficiency of energy metabolism in cardiac myocytes. In fact, this is the first study to implement a role for TGF-β_1_ in influencing mitochondrial energy metabolism in the heart. As expected, the inhibition of TGF-β signaling by the use of sR-Fc prevented the cardiac phenotype of TGF-β_1_ transgenic mice. Interestingly, chronic β-adrenoceptor blockade was also able to reverse the morphological and functional changes of the heart, whereas blockade of the angiotensin AT_1_ receptor had no significant effect on the cardiac phenotype.

The applied model of transgenic mice overexpressing mature TGF-β_1_ represents a situation of cardiac hypertrophy with preserved LV function at rest, but diminished contractile reserve. This scenario, which is manifested clinically as dyspnea on exertion in humans, is most likely to reflect the situation during the transition from compensated hypertrophy to overt heart failure. In this model, cardiac hypertrophy was not due to hypertension since there was no difference in systemic blood pressure between wild-type and TGF-β_1_ transgenic mice. Instead, subcellular mechanisms were identified that provide a molecular explanation for the TGF-β_1_-induced cardiac alterations. These include the induction of ODC, which is required for the hypertrophic responsiveness of the heart to β-adrenergic stimulation [18], and the upregulation of myocardial UCPs, which impair the efficiency of the energy metabolism in muscle cells and thus are thought to contribute to the development of contractile dysfunction in heart failure [31], [33]. UCPs are inner mitochondrial membrane proton transporters that decrease the proton electrochemical gradient across the inner mitochondrial membrane, thereby reducing the energy force for ATP production during respiration [31].

The fact that AT_1_ receptor blockade was unable to prevent myocardial hypertrophy and dysfunction may appear surprising. However, the failure of telmisartan to impede the TGF-β_1_-induced cardiac phenotype may actually be expected from previous studies. Schultz et al. provided direct proof that the hypertrophic cardiomyocyte growth induced by angiotensin II is mediated by TGF-β_1_ *in vivo* [16]. In line with this study, it was shown by several experimental approaches that TGF-β_1_ is required for angiotensin II-induced cardiomyocyte hypertrophy as it acts downstream of the AT_1_ receptor (reviewed in [5]). Hence, in a model of TGF-β_1_ overexpression, blockade of the AT_1_ receptor is not expected to prevent cardiac hypertrophy because its downstream effector is already upregulated. Therefore, our results are consistent with previous reports and further support the concept that TGF-β_1_ acts as a downstream mediator of angiotensin II in cardiomyocyte hypertrophy and dysfunction.

While the morphological cardiac alterations that are induced by TGF-β_1_ have been described in numerous studies [4]–[6], only little information is available on the functional consequences of increased TGF-β_1_ activity in the heart. We have previously shown that overexpression of TGF-β_1_ in transgenic mice was associated with an increase of myocardial β-adrenoceptor density and the downregulation of negative regulators such as G_iα_ and βARK-1, resulting in increased contractility of the atria in TGF-β_1_ transgenic mice [13]. While the induction of a hypertrophic responsiveness to β-adrenergic stimulation and increased atrial contractility in TGF-β_1_ transgenic mice appear as logical consequences of increased β-adrenergic signaling, it is difficult to understand why the inotropic response to β-adrenoceptor stimulation in LV is oppositely affected. The increased atrial contractility is likely due to a situation of diastolic LV dysfunction, where the left atrium has to compensate for the diminished LV filling in the hypertrophied heart (as reflected by the inversed E/A ratio in humans). Furthermore, an impaired contractile reserve of the LV is frequently observed in diastolic heart failure. In contrast to ventricles, no significant influence of TGF-β_1_ on UCP2 and UCP3 gene expression was observed in atrial tissue (data not shown).

While TGF-β_1_ signaling has previously not been linked to cardiac energy metabolism, our data show that stimulation of isolated cardiac myocytes with TGF-β_1_ leads to an upregulation of UCP2 and UCP3 mRNA, and that overexpression of TGF-β_1_ in transgenic mice is associated with increased levels of UCP3 protein in cardiac mitochondria. A connection between TGF-β_1_ and UCPs was shown in other systems. For instance, TGF-β_1_ induces UCP expression in fetal rat brown adipocytes [34]. Recently, UCP2 was shown to be upregulated in an aortic regurgitation model of heart failure, and UCP3 upregulation and mitochondrial uncoupling were demonstrated in viable myocardium of chronically infarcted, failing rat hearts [35], [36]. While these investigators did not relate their findings to TGF-β_1_, the upregulation of mitochondrial UCPs correlates with an increased expression of TGF-β_1_ in chronic myocardial infarction and heart failure that was shown in several animal studies as well as in human heart (reviewed in [5], [6]).

The contractile function of the heart is dependent on a sufficient energy supply that has to be continuously adapted to the energy demand, provided by substrate utilization, oxidative phosphorylation, and ATP transfer and utilization [33]. Cardiac high-energy phosphate levels are reduced in heart failure, and they correlate with indexes of diastolic and systolic function, and with NYHA functional class and mortality in heart failure patients [33].

In our model, cardiac mitochondrial UCPs were upregulated albeit contractile function at rest was normal. The functional consequences of UCP upregulation only became evident when the isolated myocytes or mice were challenged with β-adrenoceptor stimulators, and the inotropic reserve was assessed. Hence, the energy supply appeared adequate under resting conditions, but insufficient under high work-load conditions. This correlated with an upregulation of mitochondrial UCPs. Whether UCP upregulation is adaptive or maladaptive cannot be answered from our studies and requires further investigation. One speculate can, that UCPs may act as part of an adaptive response in the hypertrophied/failing heart, mainly by decreasing ROS production and lipotoxicity [36]. However, these potential benefits are likely to be offset by increased respiratory uncoupling. The resulting inefficiency of oxidative phosphorylation causes a decline in ATP transfer during high-energy phosphate metabolism. These metabolic abnormalities may contribute to contractile dysfunction and particularly to the loss of inotropic reserve that is characteristic of hypertrophied myocardium in (diastolic) heart failure [33]. Mitochondrial uncoupling might therefore play an important role in the progression from compensated hypertrophy to overt heart failure.

In addition to TGF-β_1_, the findings presented herein indicate that the β-adrenergic system is critically involved in the regulation of UCP expression. Consistent with this idea, β-adrenoceptor agonists were shown to increase the expression levels of UCPs in L6 myotubes, adipose tissue, and the heart [37]–[39]. Likewise, some studies have indicated that the partial prevention of contractile LV dysfunction in animal models of heart failure by β-blockers such as bisoprolol was associated with an improvement of cardiac energy metabolism [40]. Here, chronic β-adrenoceptor blockade by metoprolol restored the inotropic reserve in TGF-β_1_ transgenic mice, and this was accompanied by the downregulation of the initially upregulated myocardial UCPs. The functional relevance of UCPs in this context was demonstrated by the fact that genipin, acting as a UCP inhibitor [32], restored the inotropic responsiveness to β-adrenergic stimulation in isolated cardiomyocytes and *in vivo*. This may implicate that their upregulation is critically involved in the diminished contractile β-adrenergic response in TGF-β_1_ transgenic mice. Although the data obtained in human myocardium are based on a small number of samples and therefore have to be interpreted with caution, they indicate that the above mechanisms may be relevant in humans. As shown in Figure 7, myocardial UCP3 levels were elevated in hearts from DCM patients not receiving β-blocker treatment, while this was not the case in patients receiving metoprolol. These data are consistent with recent reports which indicate that energy deficiency in heart failure is associated with increased cardiac mitochondrial UCP expression and/or activity in humans. Murray et al. reported that UCP2 and UCP3 were upregulated in myocardial samples of patients with ischemia-associated cardiomyopathy [41]. Likewise, increased UCP activity was found in patients with obesity-related diabetic cardiomyopathy [42]. These data implicate that β-blockers may act beneficially in heart failure at least in part by augmenting cardiac energy efficiency.

Taken together, our data demonstrate that TGF-β_1_ oppositely regulates the hypertrophic and contractile response to β-adrenergic stimulation in the heart, leading to a phenotype of cardiac hypertrophy and myocardial dysfunction. The impairment of the inotropic reserve in TGF-β_1_ hearts from transgenic mice is linked to an upregulation of mitochondrial UCPs which influence cardiac energy metabolism. Furthermore, our findings highlight the critical role of the β-adrenergic system in TGF-β_1_-induced cardiac phenotype and indicate that β-blockers may act beneficially in cardiac hypertrophy and dysfunction at least in part by normalizing regulatory mechanisms of cellular hypertrophy and energy metabolism.
